# The toolish hand illusion: embodiment of a tool based on similarity with the hand

**DOI:** 10.1038/s41598-021-81706-6

**Published:** 2021-01-21

**Authors:** Lucilla Cardinali, Alessandro Zanini, Russell Yanofsky, Alice C. Roy, Frédérique de Vignemont, Jody C. Culham, Alessandro Farnè

**Affiliations:** 1 Cognition, Motion and Neuroscience Lab, Istituto Italiano Di Tecnologia, Genova, Italy; 2 Integrative Multisensory Perception Action and Cognition Team - ImpAct, Lyon Neuroscience Research Center, INSERM U1028, CNRS U5292, Lyon, France; 3 University UCBL Lyon 1, University of Lyon, Lyon, France; 4 Dynamique Du LangageUMR 5596Institut Des Sciences de L’Homme, CNRS- Lyon University, Lyon, France; 5 University of Lyon II, Lyon, France; 6 Institut Jean Nicod, ENS–EHESS–CNRS, Paris, France; 7 Department of Psychology, University of Western Ontario, London, ON Canada; 8 Neuro-Immersion - Mouvement et Handicap, Hospices Civils de Lyon, Lyon, France; 9 Center for Mind/Brain Sciences (CIMeC), University of Trento, Trento, Italy

**Keywords:** Perception, Human behaviour

## Abstract

A tool can function as a body part yet not *feel* like one: Putting down a fork after dinner does not feel like losing a hand. However, studies show fake body-parts are embodied and experienced as parts of oneself. Typically, embodiment illusions have only been reported when the fake body-part visually resembles the real one. Here we reveal that participants can experience an illusion that a mechanical grabber, which looks scarcely like a hand, is part of their body. We found changes in three signatures of embodiment: the real hand’s perceived location, the feeling that the grabber belonged to the body, and autonomic responses to visible threats to the grabber. These findings show that artificial objects can become embodied even though they bear little visual resemblance to the hand.

## Introduction

Our body is the means through which we interact with the external world. Little would a brain achieve without a body to execute its commands and collect information about the environment through the sensory channels. Yet our body is not just any kind of input/output machine that executes actions and provides feedback. We have a “very special regard for just one body”, such that each seems to “think of it as unique and perhaps more important than any other”^1^ We are not simply aware of one body; we are aware of it as being *our own* body (i.e. we have a sense of bodily ownership)^2^.

Throughout evolution, interactions with the environment have become more and more complex and mediated by objects that humans built and used to overcome the limitations of their bodies. Tools expand motor capabilities and allow actions that would otherwise be dangerous or impossible. There is now little doubt that tools can be incorporated: many of their properties are processed in the same way as the properties of one’s limbs^3–5^. But bodily ownership is that and more^6^. It requires experiencing tools as constitutive parts of one’s own body. Though we manipulate dozens of tools during the day, could we actually feel that a fork, a toothbrush or a screwdriver belong to us in the same way our hands do? Here we investigate whether a tool can be processed as a body part not only at the spatial level (localization), but also at the physiological level (response to threats), and at the phenomenological level (feeling of ownership). For each measure, we assess the additional impact of motor experience with the tool.

Previous studies show that even ten minutes of tool-use can deeply modify the representations of both the body and the space around it^7–15^. For example, when using a long grabber tool to retrieve objects, the arm representation is updated to reflect the functional elongation of the effector. Similarly, when using pliers, digit representations change to take into account the new morphology. Tool use also modifies the visual properties of peripersonal space, recoding far space as nearer^12,16,17^, and enhancing the defensive monitoring of such space^18^. However, while these previous studies showed that tool use affects sensorimotor and spatial representations, they did not address whether it affects body ownership, that is, whether using a tool makes it *feel* more like a part of one’s own body.

Although we seem to have little doubt about the boundaries of our own body, it has been shown that it is relatively easy to induce the illusion of owning external fake body parts. This line of research originated with the seminal paper by Botvinick and Cohen describing what is now known as the Rubber Hand Illusion (RHI)^19^. In the RHI, participants are brushed on their (hidden) hand while they see a fake hand being stroked in synchrony. The temporal congruency between what is felt on their own hand and what is seen on the fake hand leads the subjects to report that the fake hand belongs to them.

Despite the ease with which such an important change in self-perception can be induced (from just a few seconds of brushing), the illusion arises only under certain conditions. First, the real and fake body parts must be synchronously brushed; introducing a delay drastically decreases the intensity of the illusion^19,20^. For this reason, asynchronous brushing has become the gold-standard control condition, even though caution is necessary in interpreting significant synchronous vs. asynchronous differences (see below). Second, the fake limb must have a posture compatible with that of the real limb, be anatomically plausible, and appear connected to the body, suggesting that the illusion is not the mere result of a multisensory integration process between what is seen and what is felt, but is also modulated by higher-level representations of body structure^21,22^. Moreover, although a discrepancy always exists between the position of the real and fake hands in the RHI setup, the vividness of the illusion decreases the further away from the body the fake hand is placed^23^. Finally, and of most relevance here, the visual resemblance between the real hand and the other brushed object has been said to be crucial. The illusion can be induced using a fake hand that is not identical to the participant’s hand (e.g., using a rubber hand that is larger or has a different skin tone) and even to transplanted hands, prostheses, and virtual avatars^6,24–26^. However, previous studies reported the illusion could *not* be induced for objects such as wooden blocks, even those shaped like hands^27,28^. It has thus been assumed that a close visual resemblance to the body part is necessary for the sense of ownership, thus preventing tools to be felt as parts of one’s body^29,30^.

This claim, however, is controversial and has been addressed in both healthy and clinical populations. First, a sense of ownership may be reported in healthy participants for a virtual effector controlled by the subject^31^ or merely a virtual balloon changing in size and color in synchrony with the movements of the participant’s hand^32^. Yet, more recently Kalckert and colleagues^33^ showed no illusion for a static balloon. In their study, they compared the illusion induced for a fake hand with the one induced for a balloon, as in the original study, using two measures: proprioceptive drift and a questionnaire. They observed a significantly stronger illusion when a fake hand was brushed compared to when a balloon was, as indicated by proprioceptive drift of the real hand toward the fake one (vs. the balloon). They also agreed with questionnaire statements about ownership of the fake hand and feeling touch referred to the fake hand, with such ratings being stronger for asynchronous than synchronous stroking. Although the balloon condition also elicited a *difference* between synchronous and asynchronous condition in the questionnaire, the absolute ratings did not reflect a sense of embodiment even with synchronous brushing. That is, on a Likert scale from 3 = ‘strongly agree’ to − 3 = ‘strongly disagree’ where 0 = neutral, scores for the balloon were never significantly higher than zero^34^.

Taken together, the evidence suggests that objects that resemble the hand—but not those that do not—can become embodied. But what if one important factor is not visual resemblance but *functional* resemblance^35^? Perhaps what matters for the sense of ownership may be what an object can *do* rather than what it *looks* like. Evidence from special populations suggests that functional resemblance could be a key factor. Although patients with severe degenerative arthritis may still perceive a visually distorted hand as their own, amputees may feel a cosmetic prosthesis as extraneous despite its visual similarity to a real hand^36,37^. This may relate to the reasons why patients with somatoparaphrenia deny that their own hand belongs to them. They usually present with proprioceptive deficits and the so-called ‘alien’ hand is, in most although not all cases, generally paralyzed^38^: it can no longer either sense or do what the limb normally does. The functional criterion is in line with the hypothesis according to which one experiences as one’s own any part that is incorporated into the body schema, that is, into the sensorimotor representation of the body used for action^24^.

To test whether functional similarity is sufficient for embodiment, previous studies used active paradigms where the illusion was induced not by synchronous brushing, but rather by synchronous movement of the participant’s hand and an object. These studies^18,31,39^ show that such versions of the illusion can induce changes in action-related body parameters like perception of body location or ownership of a movement (agency), but other aspects of the illusion such as the conscious feeling of ownership, were not present. These results confirm that the illusion is a multilayered phenomenon that cannot be captured in its entirety by one single task. Importantly, since they did not use tools, they leave unanswered the question of whether tools can be embodied before being incorporated (i.e., before tool-use).

Because tools occupy such a pervasive place in human life, we hypothesized that it may be possible to induce the illusion that a tool is part of one’s own body even though the illusion has previously failed with other visually dissimilar objects. We further predicted that motor experience with the functional properties of the tool would modulate the expression of the illusion. Moreover, given the multifaceted nature of embodiment, we designed a series of studies to assess the three aspects of embodiment.

**Figure 1:**
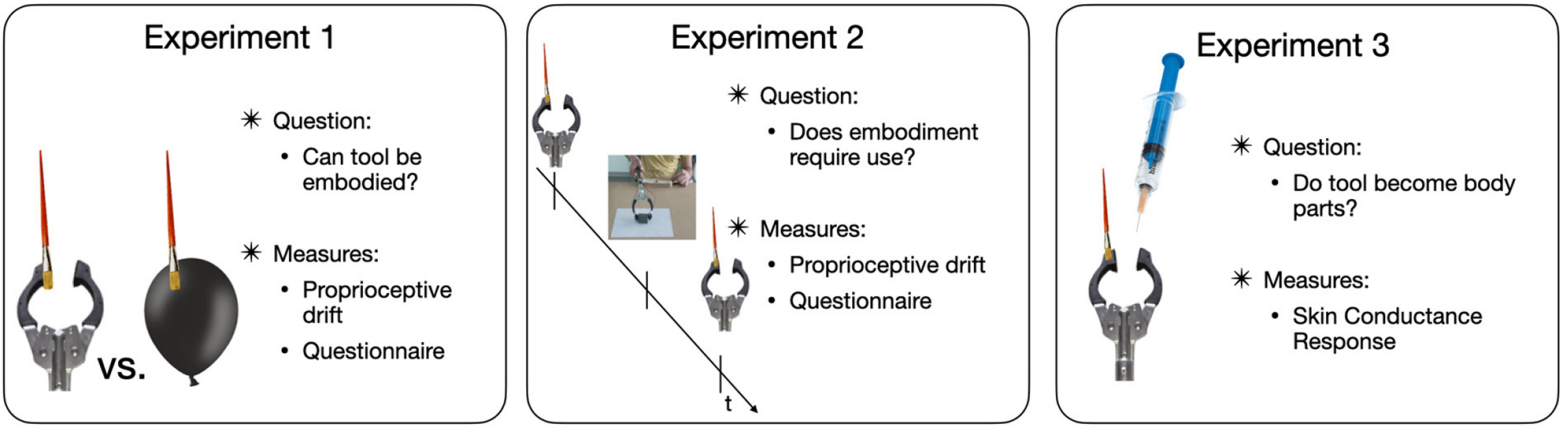
Study summary. Experiment 1 (N = 16) aimed to assess whether embodiment for a tool can be induced with an RHI-like setup. As a control, we used a balloon as in Ma and Hommel, 2015. We measured brushing-induced proprioceptive drift of the index finger and subjective feelings of embodiment using a questionnaire. Experiment 2 (N = 40) tested tool embodiment in a larger sample and investigated the role of functional use of the tool. The same measures as in Experiment 1 were used. Experiment 3 (N = 32) investigated the physiological correlates of embodiment, measuring skin conductance responses after threat to the embodied tool.

First, we applied the classic RHI paradigm by brushing the index finger of the occluded right hand of participants while they were looking either at a mechanical grabber tool being brushed or a balloon (as a control object, Experiment 1). Then, in Experiment 2 we replicated the experiment using only the grabber tool, but introduced an asynchronous condition and an active period of tool use to test the role of motor experience with the tool. Finally, in Experiment 3, we tested whether the illusion was observable via physiological responses. We measured the presence of the illusion with three different tasks (Fig. 1): (1) proprioceptive drift (to determine whether the real hand was perceived as closer to the tool after the illusion was induced), (2) a questionnaire regarding the conscious sense of ownership in (Experiment 1 and 2); and (3) a measure of arousal (Skin Conductance Response, SCR^18,40^) to a threat toward the tool in Experiment 3. Each task assesses one aspect of embodiment, defined as: “Embodiment: E is embodied if and only if some properties of E are processed in the same way as the properties of one’s body”^6^. In particular, one of those aspects is the conscious feeling that our body belongs to us (ownership), which can be assessed with questionnaires. A second aspect is the feeling that we are where our body is (which we address here with the proprioceptive drift, a measure derived by the judgement of one’s position in space). The third aspect is the physiological correlates of embodiment, measured with SCR.

In addition, we examined whether there was a perceived relationship between the digits of the hand and the prongs of the tool and if it was affected by using the tool. Typically, the tactile version of the RHI is digit-specific^41^; that is, it only occurs if the same digit of the rubber and real hand are stroked. However, since the grabber tool we used here only has two “digits” (prongs), it is not immediately clear how these would be perceived to correspond to the digits of the real hand. The correspondence could be based on visuospatial matching (e.g., whether the digit is on the left or right) or functional equivalence (e.g., whether the digit functions like a thumb or an index finger). To test this, during the brushing phase of Experiment 2, half of the participants observed the tool being brushed on its left prong while the other half saw the tool being brushed on its right prong and induction of the illusion occurred twice: once at the beginning of the session and once following a short period of tool use.

## Results

### Experiment 1

16 participants took part in this study (8 females, 1 left handed; age range 18–40). Participants gave written informed consent and received monetary compensation (15€).

**Figure 2:**
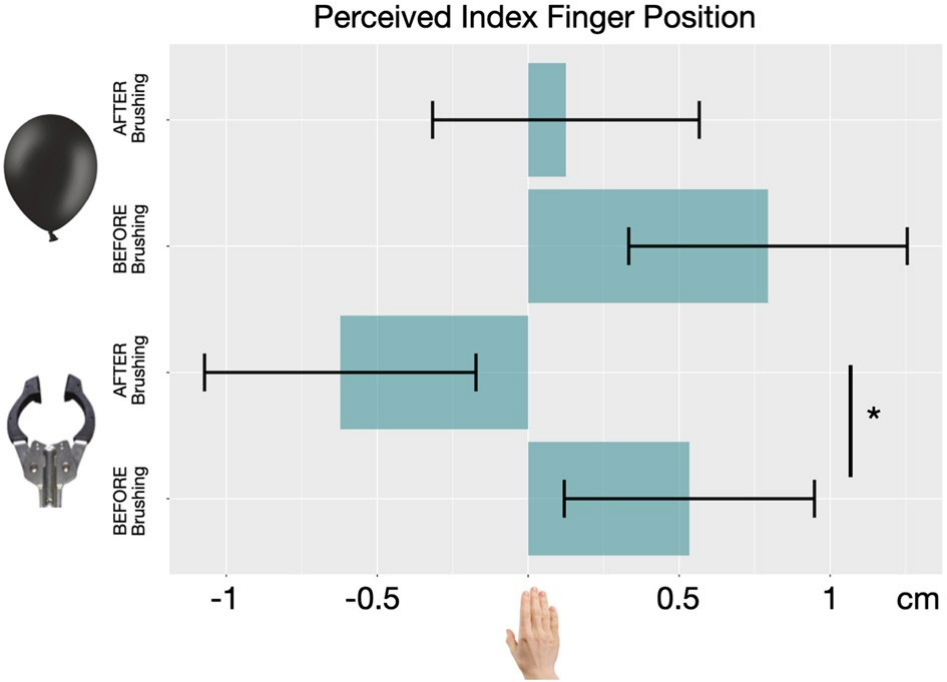
Perceived index finger position. In experiment 1, after 2 min of synchronous brushing, participants (N = 16) localised their index finger drifted toward the tool. The same participants did not show significant drift after synchronous brushing of the balloon. Error bars indicate 95% C.I.

 Proprioceptive Drift. As shown in Fig. 2 the index finger of the real hand was localized significantly closer to the tool after synchronous brushing; however, there was no significant effect drift toward the balloon. Specifically, a repeated-measures ANOVA with Object (Tool vs. Balloon) and Phase (Before vs. After Brushing) as within-subjects factors revealed a significant interaction between Object and Phase (F = 8.747, *p* = 0.01, η^2^ = 0.385). Although there was a main effect of Phase (F = 4.740, p = 0.04, η^2^ = 0.253), it was driven largely by the tool condition.

Moreover, when comparing drift amplitude (finger localization pre-brushing—finger localization post-brushing) against zero (i.e., testing for the instantiation of an illusion), we found a significant difference for the tool condition (1.2 cm; t = 2.10, *p* = 0.04) but not the balloon (0.6 cm; t = 1.69, *p* > 0.05;)*.* Finally, 63% of participants experienced an illusion (as defined by drift > 0.5 cm) with the tool compared to 37% for the balloon.

Questionnaire. Data from the questionnaire were in line with results from the proprioceptive drift task and supported the presence of illusory embodiment for the tool, but not the balloon. Separate two-tailed t-tests revealed a significant difference between tool and balloon condition for four questions, concerning touch localization and ownership (Q1. “It felt as if the touch I was feeling was caused by the paintbrush touching the tool/balloon”: t = 2.981, *p* = 0.01, d = 0.770; Q2. “It seemed like I was feeling the touch in the location where I saw the tool/balloon being touched”: t = 2.335, *p* = 0.03, d = 0.603; Q5. “I felt as if the tool/balloon were my hand”: t = 2.315, *p* = 0.03, d = 0.598; Q6. “I felt as if my hand began to resemble the tool/balloon in its posture: t = 2.884, *p* = 0.01, d = 0.745). For all the remaining control questions p values ranged between 0.10 and 0.80.

### Experiment 2

We ran a second experiment (N = 40; 22 females, 2 left handed, age range 19–40 yo) to replicate the illusion on a separate sample of participants and investigate the role of motor experience on the different components of the illusion.

**Figure 3:**
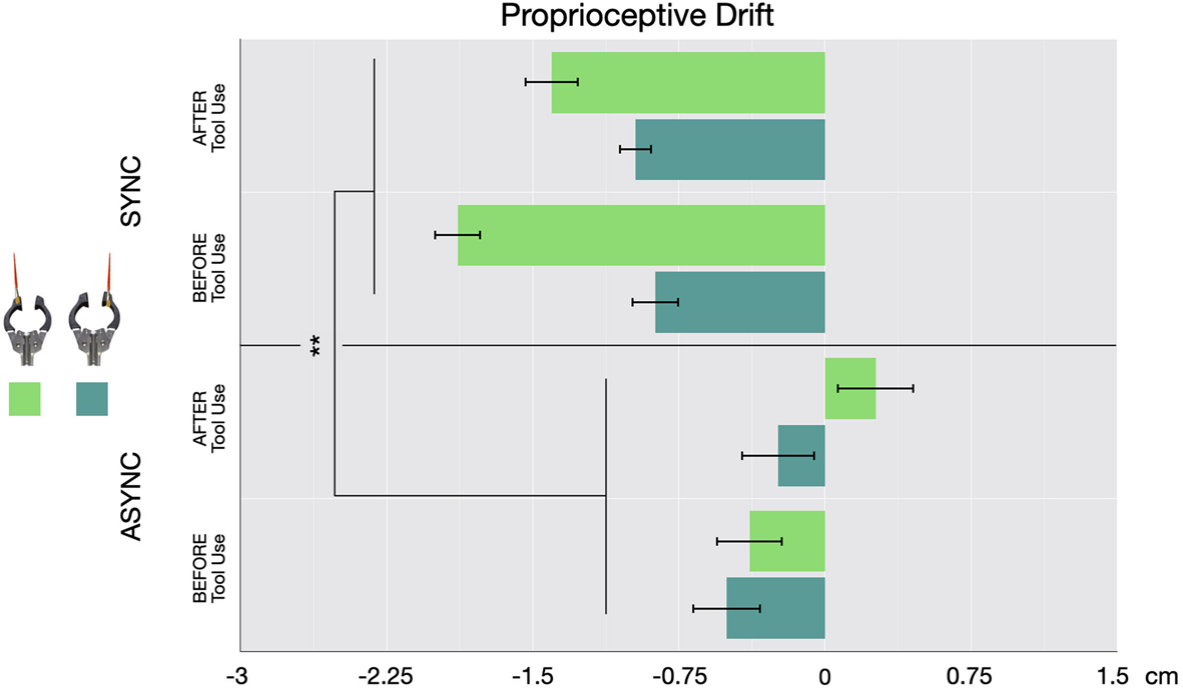
In Experiment 2, participants (N = 40) perceived their index finger drifted toward the tool only after synchronous (upper panel), but not after asynchronous (lower panel), brushing. Error bars indicate 95% C.I.

Proprioceptive drift. As shown in Fig. 3, proprioceptive drift was strongest when the tool was stroked in synchrony with hand, particularly when the left prong of the tool (compared to the right) was stroked. Specifically, an analysis of variance (ANOVA) for proprioceptive drift with three factors—Timing (Synchronous vs. Asynchronous Brushing) * Phase (Pre- vs. Post-Tool Use) as within factors and Tool Prong (Left vs. Right) as between factors revealed a main effect of Timing (stronger illusion for synchronous vs. asynchronous brushing (F = 17.919, *p* < 0.001, η^2^ = 0.131)) as well as a significant interaction of Timing*Tool Prong (F = 4.205, *p* = 0.04, η^2^ = 0.031; Fig. 3). Post-hoc t tests revealed that, while both groups showed higher drift for the synchronous compared to the asynchronous condition, the difference was greatest when the left prong of the tool was stroked. No significant difference was found between the two groups (1st prong group and 2nd prong group) when they both received synchronous stimulation (t = − 1.50; d = − 0.24; *p* = 0.3).

Tool use did not significantly affect the drift, as we observed a tendency for the drift to decrease after tool use (Phase: F = 4.27, *p* = 0.07, η^2^ = 0.012), but only for the group who saw the tool being brushed on the first prong (Fig. 3).

**Figure 4:**
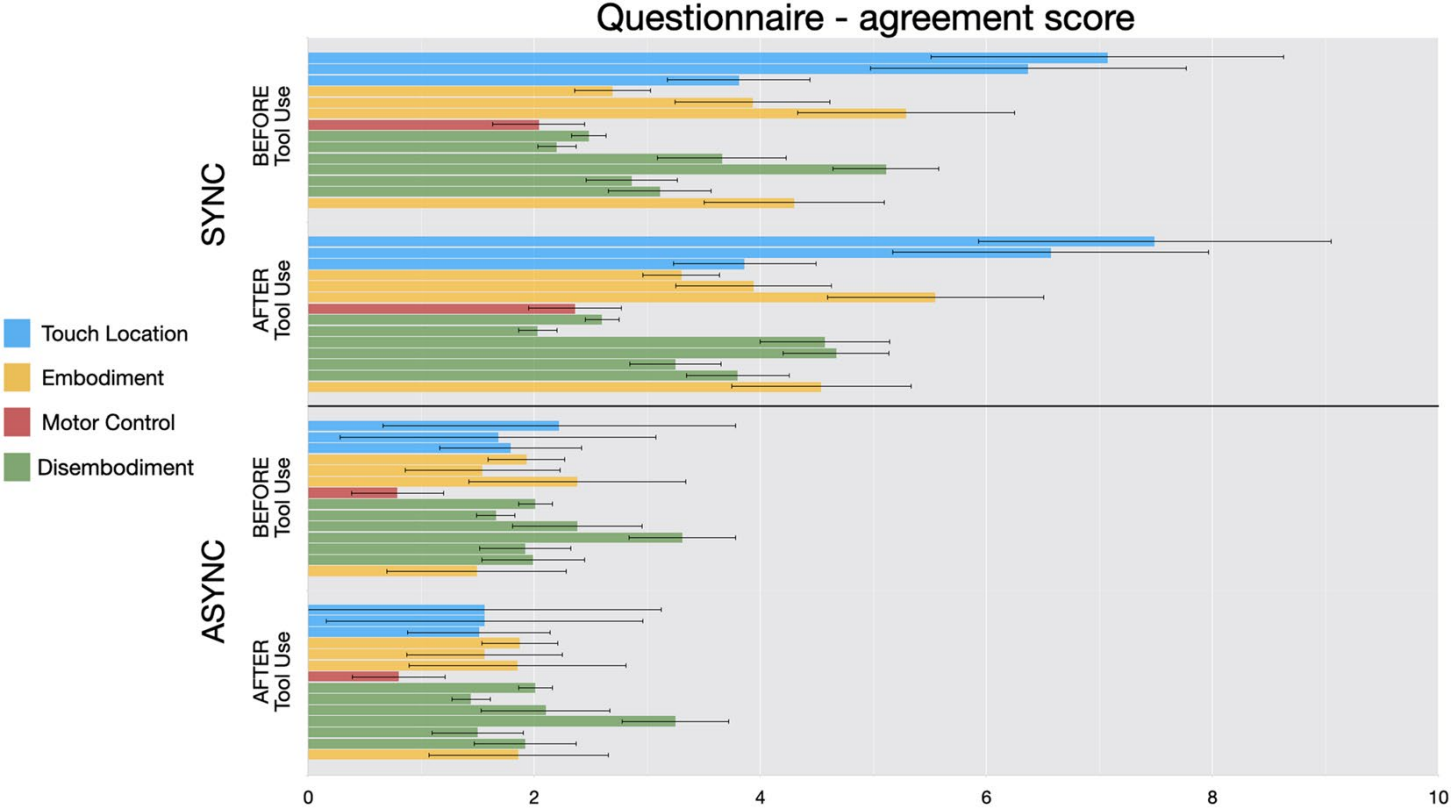
In Experiment 2, participants (N = 40) showed higher agreement for statement regarding changes in perceived touch location (Q1, Q2 and Q3 – light blue bars) and tool embodiment (Q5, Q6 and Q14 – yellow bars) after synchronous stimulation only. Error bars indicate 95% C. I.

Questionnaire. Multiple responses to the questionnaire indicated a significant degree of embodiment for synchronous stimulation, which was stronger than asynchronous stimulation. Specifically, we ran an ANOVA with Timing (Synchronous vs. Asynchronous) * Question (Q1 to Q14) * Tool Use (Before vs. After) as within factors and Tool Prong (First vs. Second) as between factor. We found a main effect of Timing (F = 53.015, *p* < 0.001, η^2^ = 0.097) showing that participants’ scores were higher (i.e. where more in agreement with a sense of embodiment) after synchronous stimulation. We also found a main effect of Question (F = 9.846, *p* < 0.001, η^2^ = 0.06) and, crucially, a significant Timing*Question interaction (F = 13.635, *p* < 0.001, η^2^ = 0.04). Post hoc tests revealed that six questions received higher scores in the synchronous condition compared to the asynchronous condition, including the four questions that were significant in Experiment 1 (Q1. “It felt as if the touch I was feeling was caused by the paintbrush touching the tool”: t = 7.217, *p* < 0.001, d = 1.141; Q2. “It seemed as if I was feeling the touch in the location where I saw the tool being touched”: t = − 9.97, *p* < 0.001, d =− 1.58; Q3. “It seemed as if I the touch I was feeling originated from a location between my hand and the tool”: t = − 4.48, *p* < 0.001, d = − 0.71; Q5. “I felt as if the tool were my hand”: t = − 4.90, *p* < 0.001 d = − 0.77; Q6. “I felt as if my hand began to resemble the tool/balloon in its posture: t = 2.884, *p* = 0.01, d = 0.745; Q14. “I felt as if the tool were part of my body”: t = − 5.64, *p* < 0.001, d = − 0.89; all p values Bonferroni corrected; Fig. 4). Additionally, we compared scores from the significative questions to “0” (corresponding to a value of 5) and found that only Q1 and Q2 were significantly higher (respectively Q1: t = 3.34, *p* < 0.001, d = 0.53; Q2: t = 1.9, *p* < 0.04, d = 0.30) and only in the Synchronous condition. All other scores where not significantly different than 0 while all items of the Asynchronous condition scored significantly lower than 0. (all *p* < 0.001).

### Experiment 3

**Figure 5:**
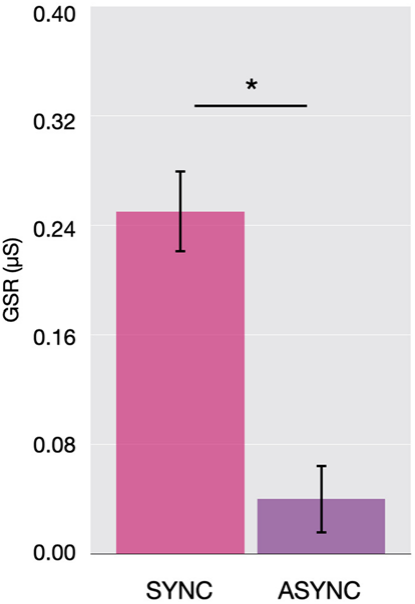
In Experiment 3, participants (N = 32) showed a Galvanic Skin Response to the threat of the tool after it was brushed synchronously, but not asynchronously, with their own index finger. Error bars indicate 95% C.I.

Experiment 3 (N = 32, 16 females, age range: 18–30 yo) was conducted to test whether proprioceptive drift and tool ownership responses reported above were also accompanied by physiological reactions that would support the idea of the tool being embodied. We recorded SCR while threatening the tool with a syringe after one minute of synchronous or asynchronous brushing. We found that participants showed a higher SCR after synchronous brushing of the hand and the tool compared to asynchronous brushing (0.25 vs. 0.04 µS; Fig. 5). However, we did not find any significant effect of experience with the tool, as the SCR did not change after tool use. Note that while the tool is threatened, the real hand is occluded by a wooden board and is 17 cm away from the tool and the syringe and thus in no ‘real’ danger. Moreover, the type of threat we used (the needle) was not actually potentially dangerous for the tool (while it could have been for the hand), which could explain why the SCR values we observed are smaller than in previous studies^42^.

## Conclusions

Here we report the first demonstration that it is possible to induce an illusory sense of ownership over a non-biological object, namely a mechanical grabber tool, that shares functional but not visual similarity with the hand. Indeed, across three experiments, synchronous brushing of the tool and the real hand induced naive participants to demonstrate that three well-established signatures of the rubber-hand illusion also occur for a tool. Specifically, induction of the illusion with synchronous stroking induced participants to: (1) localize their hand closer to where the tool was; (2) consciously report having had the experience of the tool being their hand as well as of to feel touches as coming from the tool location; and (3) show increased arousal when the tool was threatened, even though their own hand was in no danger whatsoever. Moreover, ownership was not present for a control object, a balloon, that shares neither visual nor functional similarity with the hand. Taken together, these results support our hypothesis that *functional* similarity can enable ownership over external objects for which there is no visual similarity to a hand. This result may have important implications for development of prostheses and/or wearable technologies. Although the full embodiment of augmentative technology is still highly problematic^36,37^, our data suggest that factors other than active use may favor it.

Importantly, at odds with previous work, here we found that motor experience with the tool was not necessary to experience the illusion. Previous work converged in showing that tools need to be used actively to reveal behavioral effects of tool incorporation. Specifically, several studies reported changes in arm representation only in the active tool use – when sensory feedback, mainly proprioceptive and tactile, is provided^43–45^—but not during passive holding of the tool. Interestingly, the proprioceptive information doesn’t necessarily need to arise from the tested arm. Miller and colleagues^10^, using a mirror-based setup to induce the illusory experience of controlling the tool with the left arm, showed that the representation of the left arm length was modified after active use of the tool with the right arm. Moreover, tactile recalibration was not found when the tool was only passively held by the right hand (no somato-motor feedback) or when the mirror was removed (no visual feedback). Taken together, these data suggest that when it comes to tools, the criteria for integration of sensory feedback is relatively broad and allows some discrepancies: Somato-motor information from the right arm and visual information attributed to the left arm can be combined, to update both arm representations. Perhaps the tolerance for such discrepancies also enables tool embodiment despite the gap between the real hand position and the distal end of the tool, between where the sensory receptors physically are on the skin and the tool location where sensory feedback about a movement propagates from. One could then speculate that the sensory information coming from the participants’ hand is being combined with the visual information from the touched tool, without the need of actual tool use.

Actually, here tool-use did not seem to play a major role in either establishing or modulating the illusion, which was already present before tool-use and not significantly impacted afterwards. That said, we did observe a trend: participants who looked at the tool being brushed on the first prong tended to reduce their drift after tool use, as if the second prong of the tool becomes functionally similar to the right index finger (which would be in a similar position during real-hand grasping). While further studies are needed, here we advance the possibility that two, non-mutually exclusive kinds of matching may exist between tool prongs and hand fingers (visual and functional), which might be modulated following tool use.

Taken together, these findings provide evidence for a new illusion, which we have named the “Toolish Hand Illusion”. This illusion reveals that a tool can be perceived as an owned body part, as jointly supported by three different measures, and thus different cognitive levels, of the feeling of ownership. While the present study cannot disentangle their relative weight, we suggest the Toolish Hand Illusion may not rely solely on sensory factors, such as visual similarity, but also on motor factors, such as the potential for action. To date, our findings already show that when both are removed, as in the case of the balloon condition, no ownership is observed. Our results also indicate a novel avenue for further research on the constraints of illusory body ownership, since the way the parts of a tool are mapped onto the actual parts of the body could be modulated by the experience and the perceived functionality of the tool.

## Methods

In total, 88 adult students (age range: 18–40) from Western University and Impact Lab at the Lyon Neuroscience Research Centre participated in the study (Experiment 1: N = 16, 8 females, 1 left handed; Experiment 2: N = 40, 22 females, 2 left handed, Experiment 3: N = 32, 16 females, all right handed). The number of participants to be tested was based on previous literature^31^ that addressed the question of embodiment on non-corporeal objects. Moreover, for each experiment we run a post hoc power analysis using G*Power (Faul, Erdfelder, Buchner and Lang, 2009) to check that our sample size was large enough to obtain a 0.80 power, given the effect size for the different tasks. For all three experiments, the power to detect the main effect of the stimulation was higher than 0.99.

Participants received monetary compensation ($10 for those recruited at Western University, 15€ for those recruited at CRNL). All had normal or corrected-to-normal vision and no history of neurological or psychiatric disorders and had never used the grabber before. Participants were naïve about the specific goal of the study and were fully debriefed after. All participants provided informed consent and the experiment was approved by the Psychology Ethics Board of Western University (#130319) and the French (CPP SUD EST IV #11/005) ethics committee and was conducted in accordance with the ethical guidelines presented in the revised Helsinki declaration^46^.

### Experiment 1: Tool Embodiment and role of functional similarity

The first experiment assessed whether it was possible to induce a RHI-like illusion with a grabber tool, in place of the hand, and tested the role of functional similarity between the hand and the object in driving embodiment. The grabber tool used, used in all experiments, was a mechanical grabber tool (Unger-Global, Unger Global NN400—Nifty Nabber Pro; http://www.ungerglobal.com/en/), 52 cm long in total, composed of two rubber prongs that closed symmetrically when the participant used a power grip to squeeze the handle of the tool and a 12-cm long handle. No tool use occurred in Experiment 1.

Participants were comfortably seated at a table with their right hand and arm on the table, hidden from sight under a semi-reflecting mirror. The right hand was kept in a relaxed position with right thumb close to the rest of the hand and not protruding to the side. To further reduce cues about the participant’s arm position, a large piece of black fabric was used to cover the shoulder and both upper arms. Participants were instructed to keep their left hand on their left leg.

The experiment started with a finger localization task: a ruler was placed face down over of the mirror; participants were asked to report the number on a ruler (with mm precision), corresponding to the position of their right index finger. We instructed participants to close their eyes and realign their head to the center of the body in between trials while the experimenter changed the offset of the ruler. The measure was repeated 12 times. Once the task completed and depending on the condition, a grabber tool or a black balloon were placed on the table 17 cm to the left of the participants’ right hand. The experimenter then started brushing the object on the table and the right index finger for two minutes. For the tool we brushed the left tool prong. For the balloon, which was inflated so that its width would match the one of the tool prongs, we brushed the portion of its surface that corresponded to where the left prong would be. Once the brushing completed, participants were asked to close their eyes again while the object was removed and the ruler put in place to restart the finger localization task (12 trials). The difference between finger localization measured before and after the brushing phase, called ‘Proprioceptive Drift”, was used to quantify the illusion.

Finally, participants received a questionnaire to assess their subjective experience during the illusion induction. The questionnaire was composed by 14 statements (see Supplemental Table 1 for a complete list) addressing several aspects of the illusion experience, namely “Embodiment” (Q4, Q5, Q14), “Touch Localization” (Q1, Q2, Q3); “Disembodiment of the own hand” (Q8, Q9, Q11) and “Control Questions” (Q6 and Q7). Participants were asked to rate their agreement with each statement marking a point on a line oriented from left (I don’t agree at all) to right (I completely agree).

Each participant was tested twice (once with synchronous stimulation and the other time with asynchronous stimulation) and the order of the conditions was counterbalanced across the subjects. Data from one participant were discarded as he did not complete the second session.

### Experiment 2: Tool Embodiment and role of motor experience

The experiment consisted of three phases: pre tool-use, tool-use and post tool-use. In each of the pre- and post-tool-use phases, the position of the right index finger was localized before and after illusion induction to assess proprioceptive drift and a questionnaire was administered to assess subjective experience. In the tool-use phase, participants got experience using the tool to grasp and lift objects.

Participants were seated with their right forearm resting on a table. The mechanical grabber tool was placed 17 cm to the left of the participant’s right hand such that the shaft was parallel to the participant’s forearm and the tip of the “business end” was at the same distance as the digit tips of the hand. Throughout the experiment, a small board (25 × 45 × 15 cm) occluded the right forearm from the participant’s view. To further reduce cues about the participant’s arm position, a large piece of black fabric was used to cover the shoulder and both upper arms. Participants were instructed to keep their left hand on their left leg.

During measurement of proprioceptive drift, a bigger board (100 × 55 × 20 cm) covered both the smaller board and the tool. Participants were asked to judge the felt position of their right fingertip by naming a number on a measuring stick (with mm precision) placed atop the big board above their right hand. The task was repeated six times and the origin of the measuring stick was changed in between trials to prevent judgments from being influenced by previous answers. Proprioceptive localization was measured both prior to and following induction of the illusion and the difference between them, ‘Proprioceptive Drift”, was used to quantify the illusion.

Following the measurement of proprioceptive drift, participants filled out a questionnaire: two statements focused on touch location (“It seemed like I was feeling the touch in the location where I saw the tool being touched” and “It seemed like the touch I felt was caused by the paintbrush touching the tool”), one focused on the conscious experience of the tool being one’s own hand (“I felt as if the tool were my hand”) and two served as control questions.

During illusion induction, the participant could see the distal end of the tool (from the midpoint of the shaft to the tip) but not the right hand (because the large board had been removed while the smaller board remained in place). Participants were instructed to look at the tool while it was stroked with a paintbrush either synchronously or asynchronously (depending on the session) with their own (unseen) right index finger for 2 min. They were also instructed to keep their right hand and forearm still.

Participants were randomly assigned to one of the two groups. Participants in Group 1 saw the tool being brushed on its left prong while participants in Groups 2 saw the right prong of the tool being brushed.

In the Tool-use Phase participants were asked to move to a different table where a plastic parallelepiped object (5 × 2 × 1cm) was placed 35 cm from the proximal edge of the table. On the same edge, a small colored pad served as starting point. Participants were asked to keep the fingers of the same mechanical grabber tool in contact with the colored pad and wait for an auditory instruction to start the movement (reach and grasp the object, lift, replace it and then return to the starting position). Forty-eight movements were performed.

### Experiment 3: Skin conductance response as a measure of sense of ownership

This experiment assessed an additional dependent measure of the sense of ownership: the skin conductance response to seeing the tool being stabbed by a needle.

Similarly to Experiment 2, Exp. 3 consisted of three phases (pre tool-use, tool-use, and post tool-use) but of four groups (with a factorial combination of left vs. right tool-prong and synchronous vs. asynchronous stroking). Skin Conductance Response (SCR) is a physiological measure of the electrical activity associated with increased secretions from the sweat glands resulting from sympathetic nervous system arousal. When one’s own body is threatened, skin conductance increases.

Participants were seated at a table in an anechoic room to reduce electrical interference and acoustic noise. The room was also equipped with a ventilation system that allowed to set the temperature constant (around 21.7 °C) to avoid noise in the SCR with temperature fluctuations. Room temperature was set at the beginning of each session and the experimenter checked, at the end, that no major change happened during each session. SCR was recorded using a Biopac System MP150 (Goleta, USA). Two electrodes were attached to the tips of the right index and middle fingers. Data were recorded at 100 Hz and processed with the software AcqKnowlege 4.0 for Windows. The right hand was then positioned palm down inside a box that was opened on the experimenter’s side (as well as the participant’s side) to enable brushing of the index finger. Six blocks of 60 s of brushing (synchronous or asynchronous) were alternated with a threatening block during which the experimenter briefly (~ 2 s) stabbed one of the two fingers of the tool (depending on the group the participant belonged to) with a syringe. The beginning of the brushing and the threat were manually flagged in the SCR acquisition file.

First, the threat-induced SCR response was identified by selecting the highest peak in a 5 s time window after threat onset. Then the peak-to-peak amplitude of such response was calculated and averaged across the six trials.

## Supplementary Information

Supplementary Information.
