# Supplementary material for: The toolish hand illusion: embodiment of a tool based on similarity with the hand
Source: Sci Rep. 2021 Jan 21;11:2024. doi: 10.1038/s41598-021-81706-6 (PMC7820319; doi:10.1038/s41598-021-81706-6)
Supplement: Supplementary file 1 — Supplementary Information. [file 41598_2021_81706_MOESM1_ESM.pdf]

Supplementary Table 1

The Toolish Hand Illusion: Embodiment of a tool based on similarity with the hand

Lucilla Cardinali[1*], Alessandro Zanini[2,3], Russell Yanofsky[1], Alice C. Roy[4,5], Frédérique de Vignemont[6], Jody C. Culham[7], Alessandro Farnè[2,3,8,9]

1 Cognition, Motion and Neuroscience lab, Istituto Italiano di Tecnologia, Genova, Italy

2 Integrative Multisensory Perception Action & Cognition Team - ImpAct, Lyon Neuroscience Research Center, INSERM U1028, CNRS U5292, Lyon, France

3 University UCBL Lyon 1, University of Lyon, France

4 Dynamique Du Langage UMR 5596 CNRS- Lyon University, Institut des Sciences de l'Homme, Lyon, France

5 University of Lyon II, Lyon, France

6 Institut Jean Nicod, ENS–EHESS–CNRS, Paris, France

7 Department of Psychology, University of Western Ontario, London, Ontario, Canada

8 Hospices Civils de Lyon, Neuro-immersion - Mouvement et Handicap, Lyon, France.

9 Center for Mind/Brain Sciences (CIMeC), University of Trento, Italy.

1.	J’ai eu l’impression que la sensation de touché que je ressentais était provoquée par le pinceau caressant le ballon/outil<br>*It felt as if the touch I felt was caused by the brush stroking the balloon/tool.*
2.	J’ai eu l’impression que je sentais le contact du pinceau à l’endroit où se trouvait le ballon/outil<br>*It felt as if I was feeling the touch where the balloon/tool was.*
3.	J’ai eu l’impression que la sensation de touché que je ressentais trouvait son origine entre ma propre main droite et le ballon/outil<br>*It felt as if the touch I was feeling originated somewhere in between my right hand and the balloon/tool.*
4.	J’ai eu l’impression d’avoir le ballon/outil en plus de deux mains<br>*It felt as if I owned the balloon/tool as well as my hands.*
5.	J’ai eu l’impression que le ballon/outil était ma main (faisait partie de mon corps)<br>*It felt as if the balloon/tool was my hand (belonged to my body)*
6.	J’ai eu l’impression que ma main commençait à ressembler au ballon/outil dans sa posture.<br>*It felt as if my hand posture started resembling the balloon/tool*
7.	J’ai eu l’impression que ma main droite commençait à devenir baudruche/métallique<br>*It felt as if my right hand was becoming “rubbery”/metallic*
8.	J’ai eu l’impression que ma main droite ressentait moins le touché du pinceau<br>*It felt as if my right hand could feel the touch of the paintbrush less.*
9.	J’ai eu l’impression que ma main droite n’était pas la mienne<br>*It felt as if my right hand wasn’t mine*
10.	J’ai eu l’impression que je n’étais pas capable de bouger ma main droite<br>*It felt as if I couldn’t move my right hand.*
11.	Je ne pouvais pas dire exactement où ma main droite se trouvait<br>*I couldn’t tell where my right hand was exactly*
12.	J’ai eu l’impression que ma main droite avait disparu<br>*It felt as if my right hand had disappeared*
13.	J’ai eu l’impression que ma main droite était hors de mon contrôle<br>*It felt as if my right hand was out of my control*
14.	J’ai eu l’impression que le ballon/outil faisait partie de mon corps<br>It felt as if the balloon/tool was part of my body
